# A Comparative Profile of the Burden of Human Metapneumovirus, Respiratory Syncytial Virus, and Influenza in the HIVE Cohort, 2010–2022

**DOI:** 10.1093/infdis/jiaf113

**Published:** 2025-07-16

**Authors:** Sarah S Bassiouni, Joshua E Foster-Tucker, Amy P Callear, Elie-Tino Godonou, Matthew Smith, Emileigh Johnson, Emily T Martin, Arnold S Monto

**Affiliations:** Department of Epidemiology, School of Public Health, University of Michigan, Ann Arbor; Department of Epidemiology, School of Public Health, University of Michigan, Ann Arbor; Department of Epidemiology, School of Public Health, University of Michigan, Ann Arbor; Department of Epidemiology, School of Public Health, University of Michigan, Ann Arbor; Department of Epidemiology, School of Public Health, University of Michigan, Ann Arbor; Department of Epidemiology, School of Public Health, University of Michigan, Ann Arbor; Department of Epidemiology, School of Public Health, University of Michigan, Ann Arbor; Department of Epidemiology, School of Public Health, University of Michigan, Ann Arbor

**Keywords:** cohort studies, human metapneumovirus, influenza, respiratory syncytial virus

## Abstract

**Background:**

Human metapneumovirus (HMPV) was initially identified as a cause of lower respiratory illness in young children; its characteristics are well documented in this group. However, when compared with other major respiratory viruses, less is known about its impact on adults, especially in community settings.

**Methods:**

Since 2010, the HIVE (Household Influenza Vaccine Evaluation) study has followed a cohort of households in Ann Arbor, Michigan, for acute respiratory illness (ARI) of viral etiology. Relative frequency of HMPV, respiratory syncytial virus (RSV), and influenza A and B virus (IAV/IBV) detection and associated reported symptoms was calculated; linear and Poisson mixed-effects models assessed measures of ARI burden. Additionally, a self-controlled case series analysis was conducted to characterize HMPV symptoms and burden.

**Results:**

Throughout 2010–2011 to 2021–2022, 11 714 specimens were collected. HMPV was identified in 3.4% of all specimens, RSV in 4.4%, and IAV and IBV in 6.7% and 2.5%, respectively. HMPV and RSV occurrence was comparable between adults aged 18 to 39 and ≥40 years. Mean HMPV symptom burden and severity fell between that of RSV and influenza ARIs, with HMPV associated with a rate of moderate to severe symptoms 11% below that of influenza ARI (rate ratio, 0.89; 95% CI, .79–.99). HMPV-associated disruptions in work attendance and productivity were comparable to those of RSV. No significant differences in symptom frequencies during HMPV ARIs were observed when compared with RSV, IAV, and IBV by self-controlled case series analysis.

**Conclusions:**

HMPV was comparable to RSV but not influenza in its overall occurrence, symptom frequency, and ARI burden among younger and older adults.

With the notable exception of influenza, many viruses now familiar as causative agents of respiratory infections were identified approximately 60 years ago [1–3]. They were initially identified in clinical studies, followed by a series of community-based investigations that described their frequency and other characteristics in the general population [1, 4–7]. In contrast, human metapneumovirus (HMPV) was identified much later [8, 9]. As a result, there are gaps in the full understanding of HMPV infection patterns, especially milder infections observed in community settings that do not prompt medical care.

Since HMPV was initially identified in severe respiratory illnesses among young children, its epidemiologic and clinical features among that segment of the population have historically been better characterized than in adults [10, 11]. Attention is now being directed to adults to determine whether interventions are needed for their own protection or to reduce community transmission [12, 13]. Influenza and respiratory syncytial virus (RSV) are examples of how recommendations for preventive approaches have developed over many years, leading to age-specific recommendations [14, 15]. Thus, RSV and influenza are useful comparisons when systematically evaluating the overall and age-specific impact of HMPV in a community setting.

The Household Influenza Vaccine Evaluation (HIVE) study was established in 2010, 9 years after HMPV was first identified. HIVE was developed to focus on influenza viruses but quickly expanded to the study of other respiratory viruses [16]. In the first years of HIVE, the study of households with children was conducted only to cover the broad influenza season but was expanded to year-round surveillance in 2015 [16, 17]. The focus on influenza viruses resulted in an understanding of transmission and vaccine effectiveness in the cohort, which has helped to inform vaccine policy [18, 19]. HMPV and RSV cases have been identified since the start of HIVE, in parallel with influenza. We report here on the contribution of HMPV to the burden of respiratory illnesses over the 12 years of the HIVE study, which includes 2 pandemic years. We focus on the impact of HMPV as compared with RSV and influenza A and B viruses (IAV and IBV) during the most recent 5 years.

## METHODS

### Study Population and Data Collection

HIVE is a prospective cohort study of households with children living in the region surrounding the University of Michigan School of Public Health. HIVE actively identifies acute respiratory illnesses (ARIs) that meet the study's case definition and collects a nasal specimen at onset of symptoms. Study design, laboratory methods, and consent procedures have been described previously, including adaptations made in response to SARS-CoV-2 restrictions [16, 17]. The present analysis used data collected during study years 2010–2011 to 2021–2022; see Supplementary Figure 1 for a timeline of cohort methodology.

### Statistical Analysis

#### Analysis of Relative Frequency of HMPV, 2010–2022

The relative frequencies of HMPV, RSV, IAV, and IBV were calculated by dividing the number of specimens with an identified virus by the total number of ARI specimens collected during the study as well as each study year and by age group.

#### Analysis of HMPV Burden Among Adults, 2017–2022

Participant characteristics and measures of illness burden among participants ≥18 years old were described by virus. For burden calculations, illness duration was defined as the number of days between the ARI onset date and the date of illness resolution or return to regular activity if illness resolution was not reported.

A combination of linear mixed-effects models (LMMs) and generalized linear mixed-effects models (GLMMs) was used, as appropriate, to examine the effect of virus (HMPV, RSV, or combined influenza) on illness outcome. All models included a random intercept at the individual level. LMM was used to assess whether the health rating on the worst day of illness (scale, 0–100) and productivity (scale, 0–100) during regular activities differed by virus. Mixed-effects Poisson regression was applied to count outcome variables, specifically for symptom burden and the number of sick days (illness duration). We applied a conservative estimate of symptom burden [20] to create the symptom burden outcome—a composite score—by examining only symptoms rated as moderate or severe. Hence, this composite score indicates the total number of moderate or severe symptoms per illness. Mixed-effects logistic regression was used for binary outcomes, including school or daycare missed, work missed due to illness, health care sought, any over-the-counter medications used, and a prescription received for antivirals or antibiotics. The models were implemented via the lme4 R package [21]. LMMs were fitted by restricted maximum likelihood, and GLMMs were fitted by maximum likelihood (Laplace approximation), as applied by lme4 [21]. In the LMMs and GLMMs, the reference category for the predictor was influenza. Post hoc analyses were conducted for each model to perform pairwise comparisons via the Tukey method.

A self-controlled case series analysis was conducted with conditional logistic regression to assess the association between ARI symptoms and HMPV exposure. The outcome of interest was ARI symptoms, which were assessed individually. This method compares exposure and nonexposure periods within the same individual over time (ie, individuals serve as their own controls) [22, 23]. A 1-to-1 pairing was performed, with the exposure period corresponding to an HMPV illness and the nonexposure period to an influenza or RSV illness. The closest periods in time were selected for pairing. After pairing, the time difference between closest viral infections (HMPV vs. influenza or RSV) ranged from 0.4 to 50.5 months, with a mean of 10.1 months and median (IQR) 7.8 months (2.2–13.2).

Statistical analyses were performed with R version 4.3.2 (R Foundation for Statistical Computing) and SAS software version 9.4 (SAS Institute).

## RESULTS

During the study period, participants reported 12 881 ARIs, and 90.9% (n = 11 714) had an associated nasal/oropharyngeal specimen collected for testing. Data were obtained from a total of 2703 participants with ≥1 HMPV, RSV, IAV, and/or IBV infection during the 12 study years included in this analysis (Table 1). Adults (≥18 years old) represented 42.1% (n = 1159) of the participants, with 0.3% (n = 13) of participants being ≥65 years old. For each etiologic agent, participants <18 years of age had more virus detections as compared with those aged ≥18 years.

**Table 1.** Characteristics of HIVE Cohort Participants With Histories of ≥1 Infection With HMPV, RSV, IAV, and/or IBV During 12 Study Years of Surveillance, 2010–2022

Characteristic	Overall	HMPV	RSV	IAV	IBV
Participants^a^	2703	346	466	685	278
Female	1269 (47.0)	168 (48.6)	217 (46.6)	350 (51.1)	144 (51.8)
Non-White	529 (19.6)	61 (17.3)	77 (16.5)	145 (21.2)	40 (14.3)
Age, y					
<18	1564 (57.9)	256 (74.0)	356 (76.4)	437 (63.8)	215 (77.3)
18–39	533 (19.7)	43 (12.4)	63 (13.5)	114 (16.6)	20 (7.2)
≥40	606 (22.4)	47 (13.6)	47 (10.1)	134 (19.6)	43 (15.5)

Data are presented as No. (%). Denominators are column participant totals.

Abbreviations: HIVE, Household Influenza Vaccine Evaluation; HMPV, human metapneumovirus; IAV, influenza A virus; IBV, influenza B virus; RSV, respiratory syncytial virus.

^a^Participants with a history of ≥1 infection with the given virus during follow-up.

### Relative Frequency of HMPV Overall and by Age Group in the HIVE Cohort, 2010–2022

Overall, HMPV was responsible for 3.4% (n = 393) of all ARIs with specimens collected, similar to the overall relative frequency of RSV (4.4%, n = 520; Table 2). By comparison, IAV and IBV were detected in 6.7% (n = 789) and 2.5% (n = 298) of ARI specimens, respectively. HMPV was codetected alongside RSV, IAV, or IBV in 9.1% (n = 36) of all HMPV infections, with codetection alongside RSV (n = 15) and IAV (n = 15) being equally likely.

**Table 2.** Age-Stratified Relative Frequency of Respiratory Illnesses of HMPV, RSV, and Influenza Viral Etiology Among HIVE Participants, 2010–2022

		Viral Etiology, No. (%)				
Study Period	Illnesses, No.	HMPV	RSV	IAV	IBV	FLUVs^a^
Overall						
2010–2011 to 2019–2020	9860	367 (3.7)	493 (5.0)	759 (7.7)	293 (3.0)	1052 (10.7)
2020–2021 to 2021–2022	1854	26 (1.4)	27 (1.5)	30 (1.6)	0 (0)	30 (1.6)
2010–2011 to 2021–2022	11 714	393 (3.4)	520 (4.4)	789 (6.7)	298 (2.5)	1082 (9.2)
Age, y						
≤18						
2010–2011 to 2019–2020	6147	280 (4.6)	381 (6.2)	491 (8.0)	234 (3.8)	721 (11.7)
2020–2021 to 2021–2022	1122	19 (1.7)	25 (2.2)	21 (1.9)	0 (0)	21 (1.9)
2010–2011 to 2021–2022	7269	299 (4.1)	406 (5.6)	512 (7.0)	234 (3.2)	742 (10.2)
18–39						
2010–2011 to 2019–2020	1945	39 (2.0)	64 (3.3)	125 (6.4)	21 (1.1)	145 (7.5)
2020–2021 to 2021–2022	368	6 (1.6)	1 (<0.5)	3 (0.8)	0 (0)	3 (0.8)
2010–2011 to 2021–2022	2313	45 (1.9)	65 (2.8)	128 (5.5)	21 (0.9)	148 (6.4)
≥40						
2010–2011 to 2019–2020	1768	48 (2.7)	48 (2.7)	143 (8.1)	43 (2.4)	186 (10.5)
2020–2021 to 2021–2022	364	1 (<0.5)	1 (<0.5)	6 (1.6)	0 (0)	6 (1.6)
2010–2011 to 2021–2022	2132	49 (2.3)	49 (2.3)	149 (7.0)	43 (2.0)	192 (9.0)

Rows are groups by periods before, during, and after reduced circulation of respiratory viruses following the emergence of SARS-CoV-2. Percentage positivity was calculated by using the row's illness total as the denominator.

Abbreviations: ARI, acute respiratory illness; FLUVs, influenza viruses; HIVE, Household Influenza Vaccine Evaluation; HMPV, human metapneumovirus; IAV, influenza A virus; IBV, influenza B virus; RSV, respiratory syncytial virus.

^a^ *FLUVs* summarizes influenza viruses as mutually exclusive identification of IAV or IBV.

HMPV was responsible for a larger share of ARIs among children than adults before and during the pandemic; this was also the case with RSV and IBV (Table 2). Nevertheless, HMPV was responsible for a comparable relative frequency of illnesses as RSV among adults aged ≥40 years in the decade preceding the pandemic, with both responsible for 2.7% (n = 48) of ARIs. The relative frequency of HMPV in adults aged ≥40 years was comparable to that observed among younger adults (18–39 years old), which was mirrored by infection frequencies with RSV.

### Analysis of HMPV Symptoms and Burden Among Adults in the HIVE Cohort, 2017–2022

For mean total symptoms and mean aggregate symptom severity, adults with HMPV reported slightly higher mean total symptoms and mean aggregate symptom severity than those with RSV (Table 3). Adults infected with HMPV had more frequent coughing, wheezing, and nasal congestion as compared with RSV. Adults with HMPV also cited more frequent fatigue, body aches, and fever than with RSV; these frequencies were comparable to those of adults infected with influenza who had not been vaccinated. The median illness duration for HMPV (6.0 days; IQR, 5.0–8.0) was the same as adults infected with and vaccinated against influenza (6.0 days; IQR, 4.0–8.0). Medically attended visits, encompassing outpatient visits and virtual or phone care, were infrequent for HMPV (n = 3, 9.0%) and RSV (n = 1, 2.9%) and higher for influenza infections, regardless of vaccination status (n = 23, 18.1%). In this group, only 1 hospitalization was reported, in which influenza was detected in an unvaccinated participant.

**Table 3.** Characteristics of Respiratory Virus Infection Burden Among HIVE Study Participants Over 5 Consecutive Respiratory Virus Seasons, 2017–2018 to 2021–2022

	Illness in HIVE Adults (≥18 y), 2017–2022			
			Influenza	
Characteristic	HMPV	RSV	Unvaccinated	Vaccinated
Illnesses	33	35	31	96
Age, y, mean (SD)	39.2 (7.3)	36.3 (5.2)	38.9(8.4)	42.3 (8.6)
Missed work due to illness	14 (42.4)	11 (31.4)	8 (25.8)	53 (55.2)
Work hours missed due to illness, median (IQR)	8.2 (0–12.0)	6.8 (0–11.5)	1.0 (0.0–20.0)	8.0 (0.0–17.8)
Illness duration, d, median (IQR)	6.0 (5.0–8.0)	8.0 (5.0–11.0)	5.0 (4.0–8.0)	6.0 (4.0–8.0)
Medically attended	3 (9.0)	1 (2.9)	4 (12.9)	19 (19.8)
Outpatient	2 (6.0)	1 (2.9)	4 (12.9)	16 (16.7)
Virtual/phone	1 (3.0)	0	0	5 (5.2)
Hospitalized	0	0	1 (3.2)	0
Prescribed antivirals	0	0	2 (6.5)	5 (5.2)
Prescribed antibiotics	2 (6.0)	0	1 (3.2)	6 (6.2)
Over-the-counter drugs	30 (90.9)	27 (77.1)	22 (71.0)	83 (86.5)
Aspirin	1 (3.0)	1 (2.9)	2 (6.5)	5 (5.2)
Tylenol/acetaminophen	14 (42.4)	21 (60.0)	12 (38.7)	54 (56.2)
Other analgesic/antipyretic	22 (66.7)	19 (54.3)	13 (41.9)	48 (50.0)
Cough suppressant or expectorant	15 (45.5)	17 (48.6)	7 (22.6)	51 (53.1)
Decongestant	15 (45.5)	15 (42.9)	11 (35.5)	49 (51.0)
Scale of how much illness affected productivity during regular activities (1–100), median (IQR)	60.0 (25.0–72.0)	50.0 (31.0–65.0)	55.0 (19.2–71.5)	70.5 (41.8–78.2)
Health rating on worst day of illness (0, worst; 100, best), median (IQR)	55.0 (45.0–70.0)	50.0 (40,0–69.0)	52.5 (40.0–70.0)	40.0 (30.0–59.2)
≥1 household member missed work/school to provide care	0	1 (2.9)	3 (9.7))	2 (2.1)
≥1 household member with similar illness	2 (6.0)	2 (5.7)	3 (9.7)	11 (11.5)
Cough	29 (87.9)	24 (68.6)	25 (80.6)	81 (84.4)
Fever	10 (30.3)	9 (25.7)	13 (41.9)	53 (55.2)
Fatigue	26 (78.8)	25 (71.4)	23 (74.2)	79 (82.3)
Wheezing	9 (27.3)	4 (11.4)	2 (6.5)	24 (25.0)
Total symptoms, mean (SD)	6.4 (2.1)	5.4 (2.3)	5.0 (2.0)	5.3 (2.3)
Aggregate symptom severity, mean (SD)	11.1 (5.2)	8.9 (4.6)	8.1 (4.1)	9.7 (5.5)

Data are from 2017–2018 to 2021–2022 seasons and are presented as No. (%) unless noted otherwise. Symptom severity questions were introduced in the 2016–2017 season to a subset of participants, and by the 2017–2018 season, all participants were asked symptom severity questions.

Abbreviations: HIVE, Household Influenza Vaccine Evaluation; HMPV, human metapneumovirus; RSV, respiratory syncytial virus.

We evaluated the association between detected virus and symptom presence and burden using Poisson modeling. We observed that the etiologic agent itself was a statistically significant predictor of symptom burden overall (*P* < .001). An HMPV infection was associated with an approximately 11% reduction in the rate of moderate or severe symptoms as compared with influenza (rate ratio [RR], 0.89; 95% CI, .79–.99; *P* = .040; Supplementary Table 1). HMPV (RR, 0.89; 95% CI, .79–.99; *P* = .040) and RSV (RR, 0.77; 95% CI, .69–.85; *P* ≤ .001) ARIs were associated with a lower rate or lower expected average number of moderate or severe symptoms when compared with influenza, with HMPV having the lesser reduction in symptom burden relative to influenza. Post hoc comparisons showed no statistically significant difference in symptom burden between HMPV and RSV. Illness duration—measured as the number of sick days until illness resolution, a return to regular activity, or next follow-up date if neither of the former was reported—differed according to etiologic agent (*P* < .001). We found a greater duration of RSV ARIs (RR, 1.16; 95% CI, 1.07–1.25; *P* < .001) than influenza ARIs; on average, RSV cases lasted about 16% longer than influenza cases. No statistically significant differences in illness durations were noted for influenza as compared with HMPV ARIs (RR, 1.08; 95% CI, .99–1.18; *P* = .102) or HMPV vs RSV ARIs.

### Productivity Scale and Health Rating

The percentage of adult participants who missed work due to their own illness was comparable between HMPV (42.4%, n = 14) and influenza (48.0%, n = 61), which were both higher than for RSV (31.4%, n = 11; Table 3). For those who missed work, the median number of work hours missed due to illness was highest for HMPV (8.2 days; IQR, 0.0–12.0), which was comparable to influenza (8.0; IQR, 0.0–18.0), and both were higher than RSV (6.8; IQR, 0.0–11.5). In adult participants infected with HMPV, none reported having 1 or more household member missing work or school to provide care, as compared with 1 (2.9%) for RSV and 5 (3.9%) for influenza.

The median score on the productivity scale for adults infected with HMPV (60.0; IQR, 25.0–72.0) was higher than RSV (50.0, IQR 31.0–65.0) and lower than influenza (70.0; IQR, 25.0–77.5). When asked to rate their health on the worst day of their illness (0–100, with 100 being worst) and productivity (0–100, with 0 being lowest productivity), the median score for adults was highest—indicating worse health—for HMPV (55.0), with RSV and influenza being lower (both 50.0). We evaluated health rating on the worst date of illness using linear mixed effect regression. Etiologic virus was significantly associated (*P* < .001) with health rating scores assessed on the worst day of illness. On average, participants with HMPV infection reported a health rating score that was 6.23 points higher (β = 6.23; 95% CI, 2.51–9.94; *P* = .001) than those with influenza—that is, those with HMPV felt better on the worst day of illness. A statistically significant (*P* < .001) effect on productivity was also identified, with participants with HMPV ARI reporting an average score that was 8.96 points lower (β = −8.96; 95% CI, −14.14 to −3.78; *P* = .001) on the productivity scale as compared with their counterparts with influenza. Additionally, RSV (β = −9.80; 95% CI, −14.32 to −5.27; *P* < .001) and HMPV were associated with lower impairment in productivity during daily activities relative to influenza viruses, with RSV displaying the lowest impact on productivity.

### Medication Use and Health Care–Seeking Behavior

Most adult participants who were infected with HMPV, RSV, or influenza viruses reported usage of over-the-counter drugs (90.9%, 77.1%, and 82.7%, respectively), including aspirin, acetaminophen, cough suppressants or expectorants, decongestants, or other analgesics/antipyretics. For adults infected with HMPV, other analgesics/antipyretics were the most frequently reported (66.7%) among the viruses, whereas acetaminophen was the least frequent (42.4%). In terms of over-the-counter medication use, a mixed-effects logistic regression with influenza as the reference category revealed an estimated odds ratio (OR) for HMPV of 2.45 (95% CI, .65–9.30; *P* = .187).

We used a mixed-effects logistic regression to evaluate viral etiology and participants receiving an antiviral or antibiotic prescription. The virus responsible for a given ARI was significantly associated (*P* = .014) with the odds of receiving prescriptions for antivirals or antibiotics. Specifically, adults infected with HMPV had the lowest level of reported antiviral or antibiotic prescriptions when compared with influenza (OR, 0.09; 95% CI, .01–.62; *P* = .015). Individuals who tested positive for RSV also had reduced odds of being prescribed such treatments (OR, 0.17; 95% CI, .04–.78; *P* = .022). Notably, no statistically significant difference in odds of prescription receipt was observed between participants with HMPV and RSV infections.

Medically attended visits, encompassing outpatient visits and virtual or phone care, were infrequent for HMPV (n = 3, 9.0%) and RSV (n = 1, 2.9%) and higher for influenza (n = 23, 18.1%). The etiologic agent of a given ARI was not significantly linked to the likelihood of seeking health care (*P* = .201). When influenza-associated ARIs were compared with those of HMPV or RSV etiology, the odds ratios for seeking health care were 0.66 (95% CI, .38–1.17; *P* = .157) for HMPV and 0.69 (95% CI, .42–1.14; *P* = .147) for RSV.

### Self-controlled Case Series Analysis to Assess Association Between ARI Symptoms and HMPV Exposure, 2017–2022

A self-controlled case series analysis was performed that compared the occurrence of symptoms during periods with and without exposure in the same person (Table 4). While individuals had lower odds of fever, nasal congestion, nausea, diarrhea, and wheezing during HMPV illness when compared with person-level matched influenza and RSV illnesses, no statistically significant differences were found.

**Table 4.** Self-controlled Case Series Analysis of Symptoms Associated With HMPV-Associated Events

Variable	During Event	During Control Period	Odds Ratio (95% CI)
No.	55	92	—
Medically attended	13 (25)	26 (29.9)	0.82 (.34–1.97)
Fever	31 (56.4)	64 (69.6)	0.49 (.2–1.18)
Runny nose or congestion	51 (92.7)	89 (96.7)	0.46 (.07–2.87)
Cough	51 (92.7)	86 (93.5)	1.07 (.29–3.92)
Nausea	8 (14.5)	21 (22.8)	0.46 (.18–1.21)
Diarrhea	2 (3.6)	12 (13.0)	0.13 (.02–1.07)
Dyspnea	21 (38.2)	34 (37.0)	1.14 (.49–2.68)
Wheeze	10 (18.2)	20 (21.7)	0.69 (.25–1.92)

Data are presented as No. (%). Health care visit data are missing for 5 of 92 control periods and 3 of 55 primary case events. Denominators are column totals.

Abbreviation: HMPV, human metapneumovirus.

## DISCUSSION

As with RSV, the occurrence and impact of HMPV were first recognized in young children [2, 3, 7–9]. It has taken many years to recognize the importance of RSV in older individuals, and work is still underway to understand its impact on the general population. We may find history repeating itself with HMPV. Even with a limited number of older individuals, the HIVE cohort remains an important setting to determine the burden of HMPV for adult community members.

In this article, we evaluated 10 consecutive respiratory seasons of the HIVE cohort among households of similar composition before the SARS-CoV-2 pandemic. We previously established the distinct, characteristic seasonality of these viruses in our cohort and found these patterns to be consistent with those observed in other populations [1–4, 22–24]. The relative frequency of HMPV was only slightly below that of RSV overall and closely resembled that of IBV during the prepandemic years. Among the mild to moderate illnesses described here, the proportion of illnesses due to HMPV was similar when we compared adults aged <40 years with younger adults [24]. Surprisingly, we observed that illnesses with shorter duration aligned with more work hours missed and higher median reported productivity (Table 3). Further work is needed to better elucidate this, but some potential explanations may be as follows: participants who reported a longer illness duration may have been more able to work remotely due to differences in socioeconomic status or household makeup or differential levels of presentation of presenteeism.

The illness characteristics in adults who are virally infected in a community setting are not often studied. Although not as severe as influenza, HMPV ARI overall resembled that of RSV and even exceeded it in some outcomes, such as work hours missed during illness, how much the illness affected productivity during regular activities, and use of over-the-counter medications. HMPV infection likely offers only transient immunity, given that infection can reoccur throughout the life span [25], and to date, no vaccine or antiviral management is approved for HMPV. We are familiar with the economic and social burden that viral ARIs such as RSV and influenza in children and adults pose around the world [26]. In the United States alone, there is an estimated annual economic burden of >$6 billion for direct and indirect (eg, missed work) costs associated with RSV cases in adults aged ≥60 years [27]. Influenza has long been known to have even more of an impact [28]. Thus, it is quite possible that HMPV is a significant yet underrecognized contributor to annual health care spending, mortality, and illness burden broadly among adults in the United States in its own right [29–31].

While HIVE provides rich data on time frame, specimen collection, and symptom reporting, there are some key limitations to this study. One is that there is a relatively limited number of symptomatic illnesses. This makes it difficult to stratify by certain covariates as the sample sizes become too small to conduct certain multivariable analyses. Given the case definition, it is also difficult to capture asymptomatic or minimally symptomatic respiratory infections in our study population. Thus, our results may be underestimating the true burden of HMPV infection if some participants were asymptomatic or minimally symptomatic.

Additionally, the number of individuals ≥60 years of age in HIVE households was a limitation of in determining infection impact. HIVE was designed to study the occurrence of respiratory infections in those most likely to experience them—namely, those living in households with young children. Many households in the United States do not contain older adults, although that may be changing [32]. However, the relatively comparable occurrence of HMPV between the younger and older adults in this analysis suggests that some of the results may be extrapolated to older individuals. Adults in HIVE did not have many underlying conditions, although work with RSV indicates that even younger adults with underlying conditions are at higher risk for developing severe disease [33]. Thus, the estimates here likely underestimate the potential impact of vaccinating a more high-risk population. Furthermore, as with many studies that attempt to investigate health and disease over time, HIVE is not representative of the general population. Exclusion criteria were based on household composition and health care source, with the latter done to ensure the ability to identify underlying illnesses and health care utilization.

Vaccinating children as a way to prevent transmission to adults has been considered for many years and was first carried out in influenza in the context of a household study [34]. The effectiveness of the opposite strategy remains an open question: can vaccinating older adults reduce wider community transmission of respiratory virus diseases among all ages? While the impact of influenza viruses on work absenteeism was higher, the impact of HMPV was close to that of RSV, which is substantial, and merits attempts at prevention. The recent introduction of RSV vaccines for older adults presents the opportunity to investigate the potential of indirect vaccine protection to other groups conferred by reducing infection in older adults. Likewise, continued efforts to minimize HMPV spread can result in significant direct and indirect benefits to those who interact with and care for ill household members. Our findings present evidence of substantial burden of HMPV that may warrant considering a multipronged approach to reduce HMPV burden in the household and community.

## Supplementary Material

jiaf113_Supplementary_Data

## Supplementary Data

Supplementary materials are available at *The Journal of Infectious Diseases* online (http://jid.oxfordjournals.org/). Supplementary materials consist of data provided by the author that are published to benefit the reader. The posted materials are not copyedited. The contents of all supplementary data are the sole responsibility of the authors. Questions or messages regarding errors should be addressed to the author.

## Notes

***Acknowledgments***. We thank all HIVE participants, past and present.

***Financial support.*** This work was supported in part with federal funds from the National Institute of Allergy and Infectious Diseases, National Institutes of Health, Department of Health and Human Services (contract 75N93021C00015); and the Centers for Disease Control and Prevention (grants U01IP001034, R01AI097150, R56AI097150, U18IP000170, U01IP000474).

***Supplement sponsorship***. This article appears as part of the supplement “Human Metapneumovirus Disease Burden in Older Adults and Implications for Adult Vaccination Strategies,” sponsored by AstraZeneca.

## References

[1] Chanock RM, Kim HW, Vargosko AJ, et al Respiratory syncytial virus: I. Virus recovery and other observations during 1960 outbreak of bronchiolitis, pneumonia, and minor respiratory diseases in children. JAMA 1961; 176:647–53. 13692354

[2] Parrott RH, Vargosko AJ, Kimhw, Bell JA, Chanock RM. Acute respiratory diseases of viral etiology: III. Parainfluenza. Myxoviruses. Am J Public Health Nations Health 1962; 52:907–17. 14038204 10.2105/ajph.52.6.907 PMC1523067

[3] Tyrrell DA, Bynoe ML. Cultivation of a novel type of common-cold virus in organ cultures. Br Med J 1965; 1:1467–70. 14288084 10.1136/bmj.1.5448.1467 PMC2166670

[4] Monto AS, Cavallaro JJ. The Tecumseh Study of Respiratory Illness: II. Patterns of occurrence of infection with respiratory pathogens, 1965–1969. Am J Epidemiol 1971; 94:280–9. 4328569 10.1093/oxfordjournals.aje.a121321

[5] Spigland I, Fox JP, Elveback LR, et al The Virus Watch program: a continuing surveillance of viral infections in metropolitan New York families: II. Laboratory methods and preliminary report on infections revealed by virus isolation. Am J Epidemiol 1966; 83:413–35. 4286696 10.1093/oxfordjournals.aje.a120595

[6] Chanock R, Roizman B, Myers R. Recovery from infants with respiratory illness of a virus related to chimpanzee coryza agent (CCA): I. Isolation, properties and characterization. Am J Hyg 1957; 66:281–90. 13478578 10.1093/oxfordjournals.aje.a119901

[7] Chanock R, Finberg L. Recovery from infants with respiratory illness of a virus related to chimpanzee coryza agent (CCA): II. Epidemiologic aspects of infection in infants and young children. Am J Hyg 1957; 66:291–300. 13478579 10.1093/oxfordjournals.aje.a119902

[8] van den Hoogen BG, de Jong JC, Groen J, et al A newly discovered human pneumovirus isolated from young children with respiratory tract disease. Nat Med 2001; 7:719–24. 11385510 10.1038/89098 PMC7095854

[9] Monto AS, Cowling BJ, Peiris JSM. Coronaviruses. In: Kaslow RA, Stanberry LR, Le Duc JW, eds. Viral infections of humans. New York: Springer Nature, 2014: 199–223.

[10] Williams JV, Edwards KM, Weinberg GA, et al Population-based incidence of human metapneumovirus infection among hospitalized children. J Infect Dis 2010; 201:1890–8. 20446850 10.1086/652782 PMC2873123

[11] Esper F, Martinello RA, Boucher D, et al A 1-year experience with human metapneumovirus in children aged <5 years. J Infect Dis 2004; 189:1388–96. 15073675 10.1086/382482 PMC7109939

[12] Howard LM, Edwards KM, Zhu Y, et al Clinical features of human metapneumovirus–associated community-acquired pneumonia hospitalizations. Clin Infect Dis 2021; 72:108–17. 32010955 10.1093/cid/ciaa088 PMC7823075

[13] Widmer K, Zhu Y, Williams JV, Griffin MR, Edwards KM, Talbot HK. Rates of hospitalizations for respiratory syncytial virus, human metapneumovirus, and influenza virus in older adults. J Infect Dis 2012; 206:56–62. 22529314 10.1093/infdis/jis309 PMC3415933

[14] Grohskopf LA, Ferdinands JM, Blanton LH, Broder KR, Loehr J. Prevention and control of seasonal influenza with vaccines: recommendations of the Advisory Committee on Immunization Practices—United States, 2024–25 influenza season. MMWR Recomm Rep 2024; 73:1–25. 10.15585/mmwr.rr7305a1 PMC11501009 39197095

[15] Britton A . Use of respiratory syncytial virus vaccines in adults aged ≥60 years: updated recommendations of the Advisory Committee on Immunization Practices—United States, 2024. MMWR Morb Mortal Wkly Rep 2024; 73:696–702. 39146277 10.15585/mmwr.mm7332e1

[16] Monto AS, Foster-Tucker JE, Callear AP, et al Respiratory viral infections from 2015 to 2022 in the HIVE Cohort of American households: incidence, illness characteristics, and seasonality. J Infect Dis 2025; 231:795–804. 39179953 10.1093/infdis/jiae423 PMC11847950

[17] Monto AS, Malosh RE, Evans R, et al Data resource profile: Household Influenza Vaccine Evaluation (HIVE) study. Int J Epidemiol 2019; 48:1040–g. 31038700 10.1093/ije/dyz086 PMC6693804

[18] Ohmit SE, Petrie JG, Malosh RE, Fry AM, Thompson MG, Monto AS. Influenza vaccine effectiveness in households with children during the 2012–2013 season: assessments of prior vaccination and serologic susceptibility. J Infect Dis 2015; 211:1519–28. 25416812 10.1093/infdis/jiu650 PMC4462665

[19] Malosh RE, Petrie JG, Callear A, et al Effectiveness of influenza vaccines in the HIVE household cohort over 8 years: is there evidence of indirect protection? Clin Infect Dis 2021; 73:1248. 33949666 10.1093/cid/ciab395 PMC8492146

[20] Walke LM, Gallo WT, Tinetti ME, Fried TR. The burden of symptoms among community-dwelling older persons with advanced chronic disease. Arch Intern Med 2004; 164:2321–4. 15557410 10.1001/archinte.164.21.2321

[21] Bates D, Mächler M, Bolker B, Walker S. Fitting linear mixed-effects models using lme4. J Stat Softw 2015; 67:1–48.

[22] Mostofsky E, Coull BA, Mittleman MA. Analysis of observational self-matched data to examine acute triggers of outcome events with abrupt onset. Epidemiol Camb Mass 2018; 29:804–16. 10.1097/EDE.0000000000000904 PMC6167149 30080695

[23] Iwagami M, Takeuchi Y. Introduction to self-controlled study design. Ann Clin Epidemiol 2001; 3:67–73.

[24] Falsey AR, Erdman D, Anderson LJ, Walsh EE. Human metapneumovirus infections in young and elderly adults. J Infect Dis 2003; 187:785–90. 12599052 10.1086/367901

[25] Haas LEM, Thijsen SFT, van Elden L, Heemstra KA. Human metapneumovirus in adults. Viruses 2013; 5:87–110. 23299785 10.3390/v5010087 PMC3564111

[26] Alchikh M, Conrad TOF, Obermeier PE, et al Disease burden and inpatient management of children with acute respiratory viral infections during the pre-COVID era in Germany: a cost-of-illness study. Viruses 2024; 16:507. 38675850 10.3390/v16040507 PMC11054359

[27] Carrico J, Hicks KA, Wilson E, Panozzo CA, Ghaswalla P. The annual economic burden of respiratory syncytial virus in adults in the United States. J Infect Dis 2024; 230:e342–52. 38060972 10.1093/infdis/jiad559 PMC11326840

[28] Molinari NAM, Ortega-Sanchez IR, Messonnier ML, et al The annual impact of seasonal influenza in the US: measuring disease burden and costs. Vaccine 2007; 25:5086–96. 17544181 10.1016/j.vaccine.2007.03.046

[29] Walsh EE, Peterson DR, Falsey AR. Human metapneumovirus infections in adults: another piece of the puzzle. Arch Intern Med 2008; 168:2489–96. 19064834 10.1001/archinte.168.22.2489 PMC2783624

[30] Falsey AR, Walsh EE, House S, et al Risk factors and medical resource utilization of respiratory syncytial virus, human metapneumovirus, and influenza-related hospitalizations in adults—a global study during the 2017–2019 epidemic seasons (Hospitalized Acute Respiratory Tract Infection [HARTI] study). Open Forum Infect Dis 2021; 8:ofab491. 35559130 10.1093/ofid/ofab491 PMC9088513

[31] Falsey AR, Walsh EE, House SL, et al Assessment of illness severity in adults hospitalized with acute respiratory tract infection due to influenza, respiratory syncytial virus, or human metapneumovirus. Influenza Other Respir Viruses 2024; 18:e13275. 38692663 10.1111/irv.13275 PMC11062776

[32] Pilkauskas NV, Amorim M, Dunifon RE. Historical trends in children living in multigenerational households in the United States: 1870–2018. Demography 2020; 57:2269–96. 33001418 10.1007/s13524-020-00920-5

[33] Njue A, Nuabor W, Lyall M, et al Systematic literature review of risk factors for poor outcomes among adults with respiratory syncytial virus infection in high-income countries. Open Forum Infect Dis 2023; 10:ofad513. 38033988 10.1093/ofid/ofad513 PMC10686344

[34] Monto AS, Davenport FM, Napier JA, Francis T. Modification of an outbreak of influenza in Tecumseh, Michigan by vaccination of schoolchildren. J Infect Dis 1970; 122:16–25. 5433709 10.1093/infdis/122.1-2.16

