# Supplementary material for: A Comparative Profile of the Burden of Human Metapneumovirus, Respiratory Syncytial Virus, and Influenza in the HIVE Cohort, 2010–2022
Source: J Infect Dis. 2025 Jul 16;232(Suppl 1):S101–8. doi: 10.1093/infdis/jiaf113 (PMC12265056; doi:10.1093/infdis/jiaf113)
Supplement: jiaf113_Supplementary_Data [file jiaf113_supplementary_data.docx]

SUPPLEMENTAL TABLES AND FIGURES

**Supplementary Table 1**. Characteristics associated with ARI burden using regression modeling.

|  | Etiologic Agent | | |
| --- | --- | --- | --- |
|  | **Influenza virus** | **HMPV** | **RSV** |
| Dependent Variables | Reference category | Estimated coefficient | Estimated coefficient |
| Symptom burden^1^ | — | -0.12* | -0.26*** |
| Illness length (number of sick days) ^1^ | — | 0.07 | 0.14*** |
| Missed school/daycare due to illness^2^ | — | -0.73* | -0.85** |
| Missed work due to illness^2^ | Model failed to converge. | | |
| Medically attended^2^ | — | -0.41 | -0.36 |
| Any other-the-counter drug use^2^ | — | 0.9 | -0.23 |
| Prescribed antiviral/antibiotic medication^2^ | — | -2.42* | -1.78* |
| Health rating on the worst day of illness (0–100)^3^ | — | 6.23** | 7.17*** |
| Self-reported work productivity (0–100)^3^ | — | -8.96** | -9.80*** |

* p <0.05, ** p<0.01, *** p<0.001.

^1^Mixed-effects Poisson regression; ^2^Mixed-effects logistic regression; ^3^Linear mixed-effects regression

Abbreviations: ARI, acute respiratory illness; HMPV, human metapneumovirus; RSV, respiratory syncytial virus.

**Supplemental Figure 1.** Timeline of HIVE cohort methodology between 2010/2011 and 2021/2022, including surveillance periods and assays in use. Between the 2010/2011 and 2013/2014 study years, active surveillance and specimen collection were completed seasonally between fall and spring. Surveillance periods during these years were 1 October 2010 through 30 April 2011, 1 December 2011 through 10 May 2012, 1 October 2012 through 13 May 2013, and 1 October 2013 through 30 April 2014. Since 1 October 2014, surveillance has run continuously; year-round surveillance began in full with the 2015/2016 study year, with subsequent new study years starting on July 1 and ending on June 30.


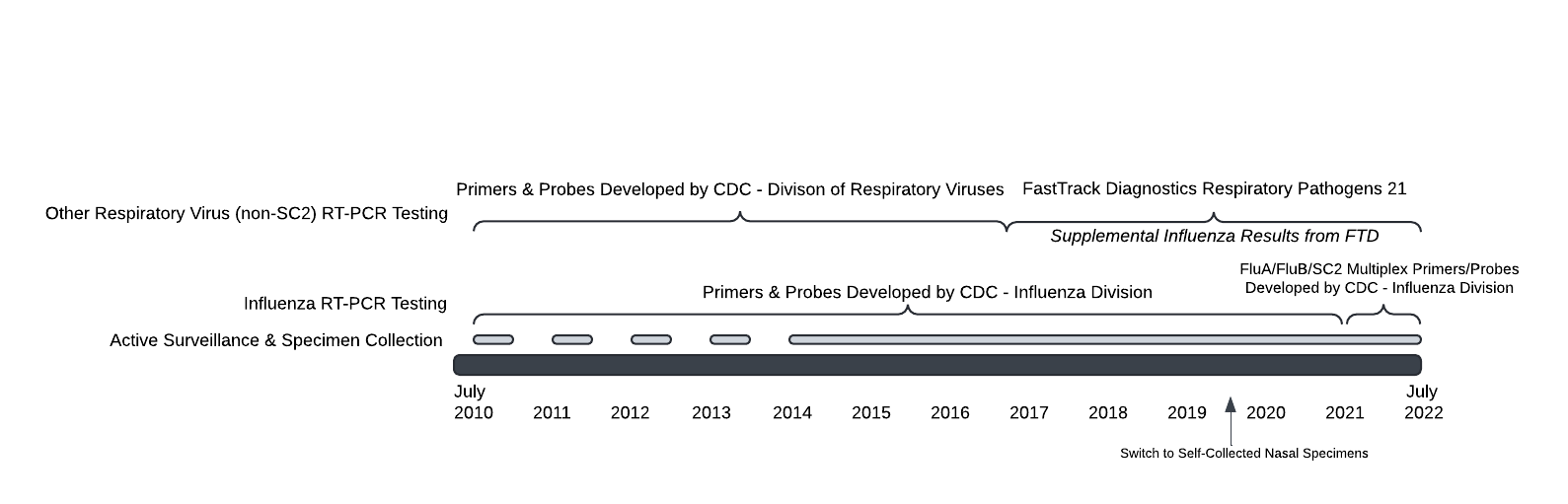


**Supplementary Figure 2**. Annual percent positivity of HMPV, RSV, IAV, and IBV among children (<18 years) and adults (≥18 years) in the Household Influenza Vaccine Evaluation (HIVE) study between 2010/2011 and 2021/2022.


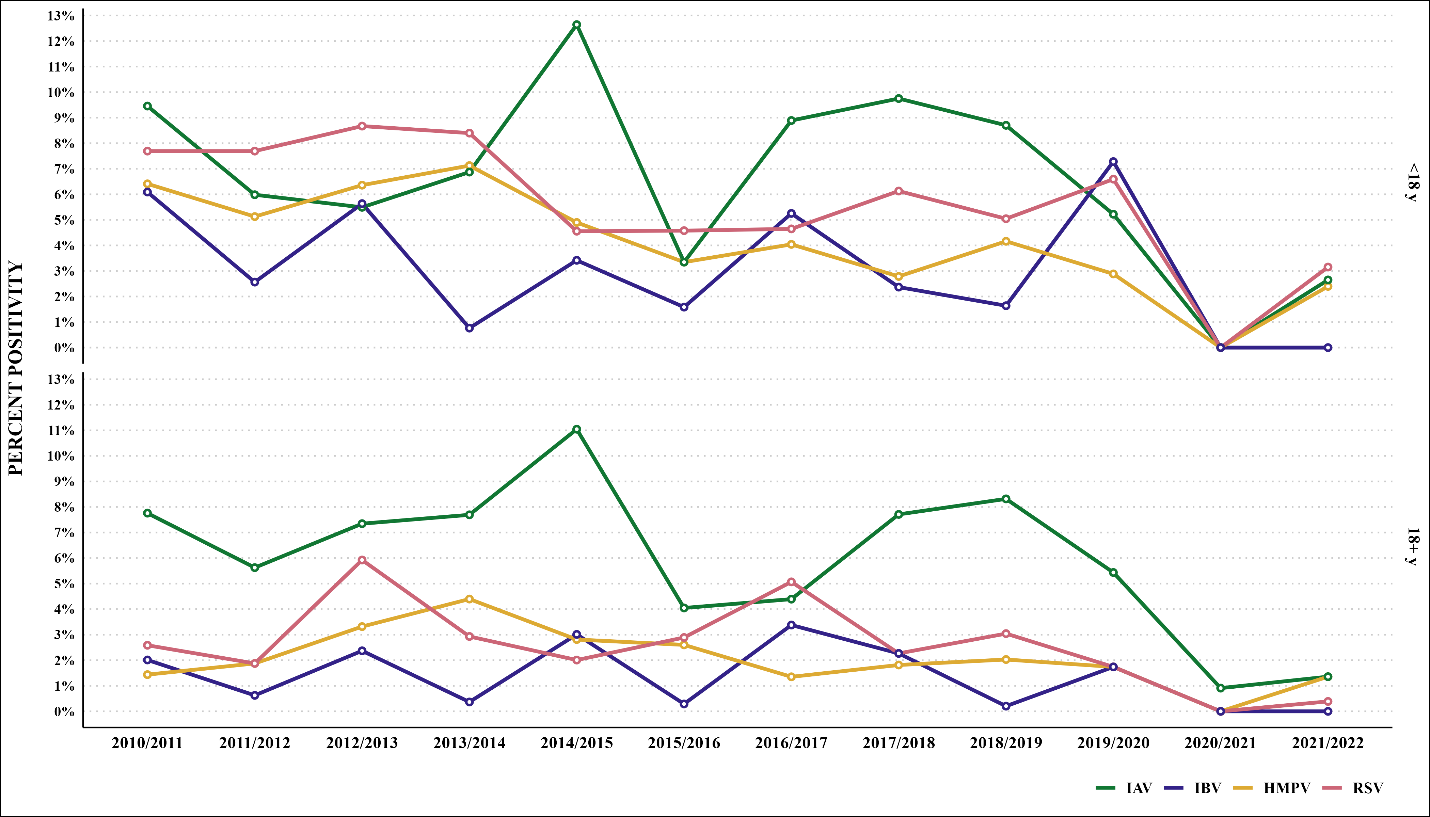


Abbreviations: HMPV, human metapneumovirus; RSV; respiratory syncytial virus; IAV, influenza A virus; IBV, influenza B virus.
